# Alcohol Abuse Mediates the Association between Baseline T/C Ratio and Anger Expression in Intimate Partner Violence Perpetrators

**DOI:** 10.3390/bs5010113

**Published:** 2015-03-20

**Authors:** Ángel Romero-Martínez, Marisol Lila, Luis Moya-Albiol

**Affiliations:** 1 Department of Psychobiology, University of Valencia, Avenida Blasco Ibañez, Valencia 21 46010, Spain; E-Mail: Angel.Romero@uv.es; 2 Department of Social Psychology, University of Valencia, Avenida Blasco Ibañez, Valencia 21 46010, Spain; E-Mail: Marisol.lila@uv.es

**Keywords:** alcohol, anger expression, intimate partner violence, testosterone/cortisol ratio

## Abstract

The imbalance between testosterone (T) and cortisol (C) levels has been proposed as a possible marker of risk for intimate partner violence (IPV). Moreover, it could be related to a high probability of adopting risky behaviors such as alcohol abuse which, in turn, promotes the onset of IPV. This study tested the potential mediating effect of alcohol consumption on the relationship between baseline T/C ratio and anger expression in IPV perpetrators and non-violent controls. Alcohol consumption was higher in the former than controls. A high baseline T/C ratio was only associated with high anger expression in IPV perpetrators, and this association was mediated by high alcohol consumption. Thus, alcohol abuse may act as a catalytic factor in this relationship, high consumption promoting the onset of IPV. These findings contribute to the development of effective treatment and prevention programs, which could introduce the use of biological markers for preventing the onset, development and recidivism of IPV.

## 1. Introduction

An imbalance between testosterone (T) and cortisol (C) levels has been proposed as a marker of propensity to violence [1]. Indeed, violent people frequently have a hypoactive hypothalamus—pituitary—adrenal (HPA) axis, which does not inhibit hypothalamus—pituitary—gonadal (HPG) axis activity. As a consequence T levels in men increase and promote the onset of violent behavior, an effect which, in turn, diminishes their sensitivity to punishment or fear, and involvement in high risk behaviors such as high alcohol intake [1,2,3].

Comparing violent with non-violent social drinkers, the former were found to have higher T levels [4], and T levels have been shown to increase after low-level acute alcohol intake [5]. Moreover, sustained higher T levels were reported in alcoholic men with no history of violence during the first week of abstinence compared to controls [6]. Similar results were found in monkeys, the administration of exogenous T increasing alcohol related-aggression [7].

On the other hand, imprisoned violent men after at least 24 hours of abstinence presented lower baseline C than unimprisoned violent men [8]. In addition, several studies have reported low baseline C levels in violent men although alcohol consumption was not considered [9,10]. In line with this, an animal study revealed that in alcohol-preferring rats high alcohol consumption was associated with high T and low corticosterone levels [11]. Hence, high T and low C levels may promote aggressive behavior after the adoption of risky behaviors such as alcohol intake. To our knowledge, however, no studies have analyzed the relationship between T/C ratio, a possible marker of risk for marital violence [3], and alcohol habits in men convicted of intimate partner violence (IPV).

Several studies have analyzed the psychological profile of the IPV perpetrators and most used a psychological approach, with little interest in biological variables and their influence in the onset and perpetuation of this kind of violence [12]. To address these lacunae in the literature, this study tested the potential mediating effect of alcohol consumption on the relationship between baseline T/C ratio and anger expression. We hypothesized that high baseline T/C ratios would be related to high anger expression in IPV perpetrators which would, in turn, be mediated by high alcohol consumption habits.

## 2. Method

### 2.1. Participants

The final sample was composed of 37 healthy male volunteers: 16 IPV perpetrators and 21 control volunteers. The IPV perpetrators were recruited from the participants in the CONTEXTO psycho-educational and community-based treatment program (mandatory for male abusers) at the Department of Social Psychology, University of Valencia. They had been sentenced to less than two years in prison and had no previous criminal record, and therefore received a suspended sentence subject to the condition that they attend this type of intervention program [13]. The experiment was performed in accordance with the Declaration of Helsinki and approved by the University of Valencia Ethics Committee (a more detailed description of the program has been published previously in Romero-Martínez *et al*. [3].

### 2.2. Procedure

Each subject participated in three sessions in the psychobiology laboratories of the University of Valencia. In the first sessions, participants were interviewed to exclude any individuals with organic diseases. The second sessions all took place between 4:00 and 7:00 p.m. After arriving at the laboratory, participants were taken to a room where they signed informed consent forms and anthropometric data (height and weight) were obtained. Moreover, participants were informed that this study did not affect their criminal report or their status in the therapeutic group. Two saliva samples were collected (for assessing T levels and C levels). Finally, in the last session, participants completed the *State-Trait Anger Expression Inventory-2* (STAXI-2), Alcohol Use Disorders Identification Test (AUDIT) and Millon Clinical Multiaxial Inventory-III (MCMI-III) alcohol dependence subscale. Finally, after this session, they completed a brief task appraisal questionnaire. Perceived stress and satisfaction were assessed using two items, and ranked on a 10-point Likert scale from 0 (low stress and dissatisfied) to 10 (high stress and satisfied). For further information see Romero-Martínez *et al.* [3,14].

### 2.3. Anger Expression

Anger expression was measured by an adapted version [15] of the *STAXI-2* [16]. This test is distributed into four subscales: anger expression out, anger expression in, anger control out, and anger control in. To reduce the number of tests, increase power for effect size, and aid interpretation within a conceptual framework, a general anger expression index (AEI) was calculated by summing the scores of the two expression subscales, subtracting the scores of the two control scales, and finally adding 36 units to avoid negative scores. The Cronbach’s alpha ranged from 0.67 to 0.89.

### 2.4. Alcohol Abuse

To assess alcohol consumption, the Spanish version of the AUDIT [17] was used. This questionnaire includes questions about the quantity and frequency of alcohol use in adults. It was developed by the World Health Organization (WHO) to identify individuals whose alcohol consumption has become hazardous or harmful to their health. It is composed of 10 self-report items with responses ranging from 0 (never) to 4 (daily or almost daily). The AUDIT is distinguished from other well-known screening instruments by the fact that items are scored on a frequency continuum (rather than dichotomously), it requests information referring to a limited time period (e.g., 6 months *vs.* lifetime), and it seems to have broader applicability in that it identifies hazardous and harmful drinkers (*i.e*., at-risk problem drinkers) rather than just those who are alcohol dependent [18]. The Cronbach’s alpha was 0.88.

In addition, the Alcohol Dependence scale of the MCMI-III [19] was used. This instrument is a self-report inventory consisting of 175 dichotomous items which measure personality disorders. It comprises 3 Modifying scales; 11 Clinical Personality Patterns scales; 3 Severe Personality scales, 7 Clinical Syndromes scales, and 3 Severe Syndrome scales. The Spanish version validation reported a reliability of between 0.65 and 0.92.

### 2.5. Hormone Measurements

Saliva was directly collected from the mouth using Salivette devices for C (Sarstedt, Rommelsdorf, Germany) and sterile glass tubes for T samples. In all cases, participants were informed about the need to follow the instructions for saliva sampling to obtain meaningful data and samples were collected in the same order, C then T, and frozen at –20 °C until analysis. Salivary T levels were assessed by chemiluminescence immunoassays using testosterone saliva ELISA kits (Diagnostics Biochem Canada) and salivary C levels by radioimmunoassay using Coat-A-Count cortisol kits (DPC-Siemens Medical Solutions Diagnostics). For more details see Romero-Martínez *et al.* [3,14].

### 2.6. Data Analysis

After confirming the normality of the data using the Kolmogorov—Smirnov test, *t*-tests with Levene’s test for equality of variances were used to check for significant differences in age, BMI (Body mass index), impulsivity and baseline T/C ratio levels between groups (IPV and controls).

To test the hypothesis that alcohol abuse mediates the relationship between baseline T/C ratio and their AEI, we conducted mediation analyses, including Preacher and Hayes’ [20] procedures for conducting a bootstrap analysis of the sampling distribution of the indirect effect.

Data analyses were performed using SPSS 21.0 (SPSS IBM) and *p* ≤ 0.05 was considered significant. Average values are expressed as mean ± SEM.

## 3. Results

### 3.1. Sample Characteristics, T/C Ratio and Anger

IPV perpetrators did not differ significantly from controls in age (38.31 ± 10.37 and 35.81 ± 6.76, respectively), BMI (26.81 ± 3.33 and 27.55 ± 2.89 Kg/m^2^, respectively), or AEI (19.83 ± 10.11 and 21.90 ± 7.82, respectively). Moreover, there was no significant difference between groups in baseline T/C ratio (1.1 × 10^−3^ ± 1.24 × 10^−3^ and 0.6 × 10^−3^ ± 0.54 × 10^−3^, for IPV perpetrators and controls, respectively). On the other hand, groups differed in AUDIT (t_35_ = 2.79, *p* = 0.008, *d* = 0.94) and MCMI-III (t_35_ = 4.51, *p* = 0.000, *d* = 1.52) scores, with IPV perpetrators obtaining higher scores for alcohol consumption than controls. Furthermore, IPV perpetrators reported to have similar appraisal scores than controls in satisfaction (5.00 ± 1.79 and 5.78 ± 1.82, respectively), but they perceived the TSST as less stressful than controls (3.41 ± 2.58 and 5.57 ± 2.38, respectively), being this difference significant (t_35_ = −2.65, *p* = 0.012, *d* = 0.90).

Is baseline T/C ratio associated with anger expression index in IPV perpetrators? Does high alcohol consumption mediate this association?

**Figure 1:**
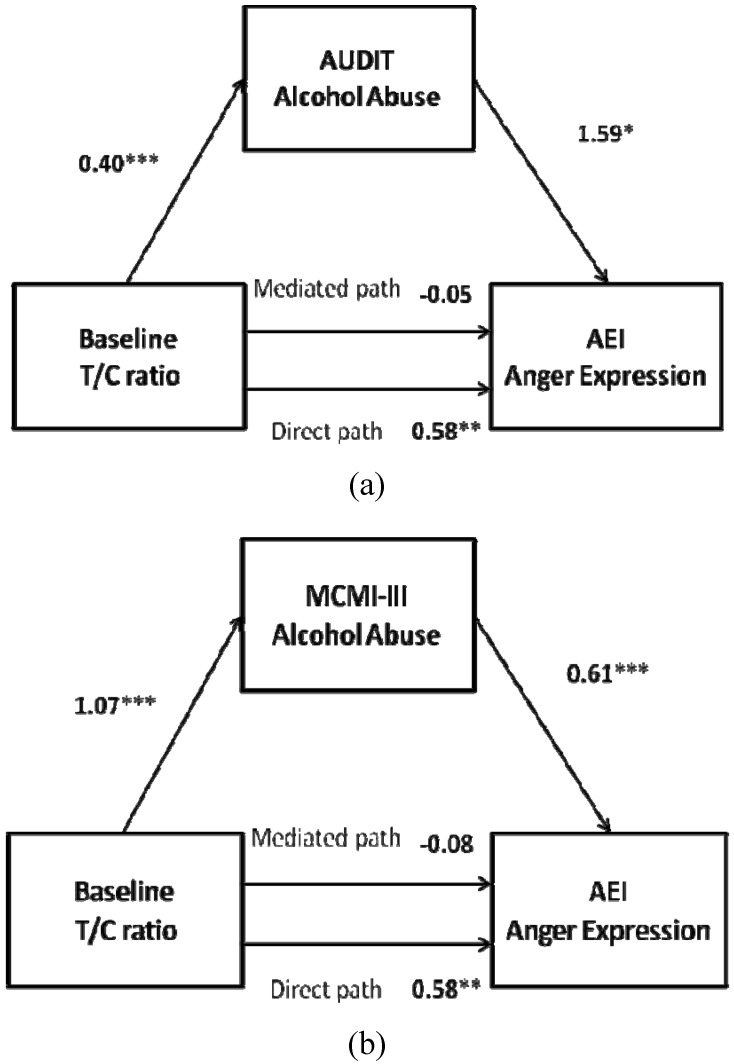
Baseline testosterone/cortisol (T/C) ratio as a predictor of (**a**) anger expression index (AEI) anger expression mediated by Alcohol Use Disorders Identification Test (AUDIT) alcohol consumption and (**b**) by Millon Clinical Multiaxial Inventory-III (MCMI-III) alcohol abuse in intimate partner violence (IPV) perpetrators. * *p* < 0.05, ** *p* < 0.01, *** *p* < 0.001.

First, considering alcohol abuse as indicated by AUDIT scores (displayed in Figure 1a), the direct effect of baseline T/C ratio on alcohol abuse (AUDIT) was statistically significant (β = 0.40, SE = 0.06, *p* = 0.000), as were the direct effects of alcohol abuse (AUDIT) and of baseline T/C ratio on their AEI (β = 1.59, SE = 0.62, *p* = 0.024; and β = 0.58, SE = 0.18, *p* = 0.006, respectively). In addition, when accounting for the effect of sensitivity to alcohol abuse (AUDIT), the effect of baseline T/C ratio on AEI reduced to nonsignificance (β = −0.06, SE = 0.29, *p* = 0.844). Results of the bootstrap analysis indicate that mediation was present (*M* = 0.65, *SE* = 0.07; 95% CI = 0.27 to 1.30). Thus, it can be concluded that the association between baseline T/C ratio and AEI in IPV perpetrators was mediated by their high alcohol abuse, in terms of the AUDIT scores. The statistical power for the analysis was 0.96.

Second, considering alcohol abuse as reflected by MCMI-III scores (shown in Figure 1b), the significant associations were similar, namely, the direct effect of baseline T/C ratio on alcohol abuse (MCMI-III) was also statistically significant (β = 1.07, SE = 0.24, *p* = 0.001), as were the direct effects of alcohol abuse (MCMI-III) on AEI (β = 0.61, SE = 0.12, *p* = 0.001) and of baseline T/C ratio on AEI (β = 0.58, SE = 0.18, *p* = 0.006). Likewise, when accounting for the effect of sensitivity to alcohol abuse (MCMI-III), the effect of baseline T/C ratio on their AEI reduced to nonsignificance (β = −0.08, SE = 0.17, *p* = 0.645). Results of the bootstrap analysis indicate that mediation was present (*M* = 0.65, *SE* = 0.61; 95% CI = 1.58 to 1.91). Thus, as with the AUDIT scores, the association between baseline T/C ratio and AEI in IPV perpetrators was mediated by their high alcohol consumption (MCMI-III). The statistical power for the analysis was 0.99.

All analyses were also carried out for controls, and there were no significant associations between T/C ratio with the AEI (β = −0.155, SE = 0.34, *p* = 0.651), alcohol abuse assessed by the AUDIT (β = 0.35, SE = 0.84, *p* = 0.678) and with the MCMI-III (β = 0.09, SE = 0.24, *p* = 0.714). Moreover, the AUDIT and the MCMI-III scores were unrelated with the AEI (β = −0.35, SE = 0.94, *p* = 0.713 and β = 0.08, SE = 0.10, *p* = 0.938, respectively).

## 4. Discussion

IPV perpetrators were more frequent consumers of alcohol than controls. High baseline T/C ratio was only associated with high anger expression in IPV perpetrators. Moreover, that relationship was mediated by high alcohol consumption as assessed by both of the instruments used (AUDIT and MCMI-III). There were, however, no differences between groups in the baseline T/C ratio levels. The observed power for the regression models was high (which ranged from 0.97 to 0.99), given the observed probability level, the number of predictors, the observed R^2^, and the sample size. Hence, we concluded there is adequate power to conduct the analyses presented.

It is important to elucidate the role of alcohol in IPV due to the fact that a high percentage of IPV perpetrators assault their partners under its effects and/or have alcohol dependence [21,22,23]. Our previous findings indicted that violent men had higher T levels and lower HPA axis response to stress than controls [14]. Thus, we proposed that the T/C ratio could be used as a risk marker for domestic violence [3]. Our current data reveal the mediating role played by alcohol in the relationship between the imbalance in T and C levels with anger expression. Specifically, an imbalance in the products of the HPG and HPA axis may predispose individuals to adopt risky behaviors such as high alcohol intake which, in turn, may promote the onset of violent behaviors.

A limitation of our study is the cross-sectional and non-experimental design which does not allow causality to be addressed. Additionally, the lack of information about the IPV perpetrators’ current severity of alcohol dependence (age of onset of dependence, alcohol consumption per day and poly-drug use) limit the external validity of our findings. Moreover, it should be noted known that the STAXI-2 tends to asses anger expression in general (including IPV). Hence, it should be convenient employ instruments, which specifically measure the IPV perpetration. For this reason, future work should include replicating these findings considering the nature of the alcohol consumption and two additional groups of men with no history of IPV, one of consumers and another of non-consumers of alcohol.

## 5. Conclusions

In conclusion, our study reveals that alcohol abuse mediates the positive relationship between baseline T/C ratio and anger expression in IPV perpetrators, but not in controls. Hence, the present data offer a wider explanation of IPV improving our understanding of the interactions of imbalances in HPG and HPA axes in IPV perpetrators with anger expression. Alcohol abuse may act as a catalytic factor in this relationship as high consumption promotes the onset of IPV. Due to the limited results obtained in psychotherapeutic programs developed for this population, it is worthwhile exploring a different approach based on biopsychosocial models that include psychobiological variables such as hormonal, immunological, psychophysiological and neuropsychological parameters. This wider perspective would contribute to the development of effective treatment and prevention programs based on the creation of better IPV perpetrators criminal profiles than the present profiles based exclusively on psychological variables. However, further confirmatory studies are needed.
